# Development of Ling-zhi industry in China – emanated from the artificial cultivation in the Institute of Microbiology, Chinese Academy of Sciences (IMCAS)

**DOI:** 10.1080/21501203.2016.1171805

**Published:** 2016-04-28

**Authors:** Saifei Li, Caihong Dong, Hua’an Wen, Xingzhong Liu

**Affiliations:** State Key Laboratory of Mycology, Institute of Microbiology, Chinese Academy of Sciences, Beijing, China

**Keywords:** Cultivation, *Ganoderma*, Institute of Microbiology, Ling-zhi, industry

## Abstract

Ling-zhi is a medicinal herb that generally refers to a fungus in the genus *Ganoderma*. It has been used as a medicinal mushroom in traditional Chinese medicine for more than 2000 years. Mycologists at the Institute of Microbiology, Chinese Academy of Sciences (IMCAS) first artificially cultivated the Ling-zhi fruiting body in the late 1960s (X.J. Liu’s team). In IMCAS, different research teams have extensively studied Ling-zhi in the aspects of national resource surveys, systematic taxonomy, chemical analysis, and processing for medicinal and health applications. The research results from IMCAS have provided essential support and prompted the development of the Ling-zhi industry in China to some extent. This review aims to summarize the history of research on Ling-zhi in IMCAS and its role in the development of the Ling-zhi economy.

## 1. Ling-zhi in ancient China

In ancient China, Ling-zhi was regarded as a magic herb called xian-cao (仙草), rui-cao (瑞草), or rui-zhi (瑞芝) for its representation of good luck and happiness. Myths worshipping Ling-zhi can be found in folk legends, and poems in praise of happiness and sanctity have been written with Ling-zhi as the symbol. The Chinese traditional handicraft ru-yi (如意) was designed from Ling-zhi, and Ling-zhi was also a common element in Chinese drawings and paintings.

The application of Ling-zhi can be traced back to 2000 years ago. It had been used for the treatment and prevention of various diseases as a famous traditional Chinese medicine, which was earliest documented in *Shen Nong Materia Medica* (Anonymous 1955). After then, many scholars in the early history had recorded Ling-zhi in different ways. The most famous one was Li Shi-Zhen (李时珍)，who had illustrated Ling-zhi as a vegetable in his *Compendium of Materia Medica*, and believed that it had the medicinal value to enhance the spirit and energy of the body.

## 2. Research on Ling-zhi

From the ancient, till now, Ling-zhi has changed from a wild fungus to a recognized macrofungus and modern Chinese medicinal material with its own industry. Many scientists from different fields have contributed to the development of Ling-zhi in China. Among which, mycologists from the Institute of Microbiology, Chinese Academy of Sciences (IMCAS) have provided significant contribution that have aided the development of the Ling-zhi industry in China. This review aims to summarize the researches on Ling-zhi by different teams from IMCAS since the 1930s, and the general development of the Ling-zhi industry in China.

### 2.1. National survey on Ling-zhi resources in China

Patouillard first named the Chinese collections from Guizhou Province as *Ganoderma lucidum* (Curtis) P. (Patouillard 1907). Then, systematical studies were carried out by mycologists from different institutes and universities in China. In IMCAS, Ling-zhi species were systematically recorded by two famous Chinese mycologists, S.C. Teng. and F.L. Tai, from the 1930s to the 1970s. Teng was first director and vice director of IMCAS and underwent the Ling-zhi scientific investigation in China in the early period. He identified four species and one variety of *Ganoderma* P. Karst. (Teng 1934). Later, he broadened the *Ganoderma* genus to include certain polyporous fungi and recognized 19 more species in his famous book *Fungi of China* (Teng 1963). In addition, 11 species in the genus *Amauroderma* Murrill, which were also generally called Ling-zhi in China, were recorded in his book. In 1979, 27 *Ganoderma* species and 11 *Amauroderma* species were recorded in *Sylloge Fungorum Sinicorum* by Tai (Tai 1979). These early records on Ling-zhi species helped people form a primary understanding of this group of fungi.

Mycologists from IMCAS also contributed the Ling-zhi popularization and cultural inheritance. Ying et al. (1987) initially described six *Ganoderma* species in *Icons of Medicinal Fungi from China*. This monograph was issued to 17 other countries and helped to popularize Chinese medicinal fungi to the world. Then, Mao (1998) described 20 medicinal *Ganoderma* species in *Economic Fungi of China*, and increased the number to 67 in *The Macrofungi in China* (Mao 2000). Moreover, the centuries-old Ling-zhi culture in China, coupled with Ling-zhi drawings, was included in different scientific popular articles, as well as professional atlas (Mao 2009). These publications better informed the public on Ling-zhi culture.

After Ling-zhi species were preliminarily studied, the taxonomy of Ling-zhi species was investigated from the 1970s through the 2000s by Zhao and his colleagues at IMCAS. Ling-zhi, including species of *Ganoderma* and other genera, was included in the family Polyporaceae because of its cavernous hymenium. Zhao et al. (1979) recognized a subfamily as Ganodermoideae Donk, stating that spore features should be emphasized in the taxonomy of these species. Fifty-three species, one variety and one form were verified in China at that time (Zhao et al. 1979). Then, in *Ling-zhi of China* (Zhao et al. 1981) and *New Ling-zhi of China* (Zhao 1989), Ganodermataceae Donk was recognized after a systematic morphological study. Subsequently, Chinese species of Ganodermoideae and Ganodermataceae were reported in series papers from 1979 to 1998 by Zhao and his colleagues (Zhao et al. 1979, 1983, 1984, 1986; Zhao & Zhang 1986, 1987a, 1987b, 1992; Zhao 1987, 1988a, 1988b; Zhang 1997, 1998). A comprehensive and systematic taxonomy of Ganodermataceae in China was monographed in *Flora Fungorum Sinicorum vol*. 18, which described four genera (*Ganoderma, Amauroderma* Murrill, *Haddowia* Steyaert, and *Humphreya* Steyaert), and 98 species in detail (Zhao & Zhang 2000).

### 2.2. Phylogenetic study

With the application of molecular techniques in modern taxonomy, the phylogeny of Ganodermataceae was then investigated by Y.J. Yao from IMCAS. Yao’s team initially found that the internal transcribed spacer (ITS) heterogeneity occurred in *Ganoderma* strains (Wang & Yao 2005). They also reconstructed the phylogeny of Ganodermataceae and its allied genera using multigene sequences including ITS, IGS, nrLSU, nrSSU, rpb2, tef1, β-tubulin, mtSSU, mtLSU, and atp6 genes, and concluded there were at least seven genera in the family Ganodermataceae, i.e. *Ganoderma, Humphreya, Tomophagus* Murrill, *Trachyderma* (Imazeki) Imazeki, *Magoderna* Steyaert, *Amauroderma*, and *Haddowia* (Wang 2012). Continuously, they extensively investigated the widely cultivated ‘Ling-zhi’, which was recognized and accepted as ‘*Ganoderma lucidum*’ in the scientific community and industry. However, the widely cultivated Ling-zhi in China is different from the *G. lucidum* species originally described from England, based on morphological observations and molecular studies. After a detailed study of the epitypification of cultivated Ling-zhi, *G. lucidum* was considered to be conspecific with *G. sichuanense*, a species that was initially described by Zhao and Zhang in 1983 (Wang et al. 2012). This result was accepted by Wu et al. (2013). While Cao et al. (2012) found that Ling-zhi represented an independent lineage, and a new species, *Ganoderma lingzhi* Sheng H. Wu, Y. Cao & Y.C. Dai, was introduced for the cultivated Ling-zhi (Cao et al. 2012; Zhou et al. 2015). In 2013, *G. lingzhi* was nominated as the fungus of the year for Mycology (Yang & Feng 2013). Moreover, the name *G. lingzhi* has been used instead of *G. lucidum* by most mycologists (Fatmawati et al. 2013; Gao et al. 2014; Li et al. 2014; Chen et al. 2015; Yan et al. 2015; Wang et al. 2016).

### 2.3. Artificial cultivation of Ling-zhi fruiting body

Another important breakthrough was the successful cultivation of the fruiting body of Ling-zhi by scientists from IMCAS. This made it possible for the wide usage of Ling-zhi resources worldwide.

In the late 1960s, a wild Ling-zhi fruiting body was presented to Chairman Zedong Mao by Unit 6037, Chinese People’s Liberation Army, as a loyalty gift. Chairman Mao transferred this Ling-zhi fruiting body to Moruo Guo, the president of the Chinese Academy of Sciences, and subsequently to the IMCAS for study. X.J. Liu, a mycologist at IMCAS was appointed to lead this programme and started research on the artificial cultivation of Ling-zhi fruiting body. Although some attempts were made before Liu’s programme, all failed because only stipes of the mushroom formed without pilei. Liu’s team found that humidity in the later stage of the fruiting process was the critical factor for fruiting body formation. High humidity (85–95%) in the fruiting body formation stage is essential for intact pileus development, while low humidity only forms a staghorn-like pileus (Anonymous 1992). This was the first successful cultivation of Ling-zhi fruiting bodies in China. The first sample of *Ganoderma* spore powder was collected and displayed in the Mycological Herbarium, Institute of Microbiology, Chinese Academy of Sciences (HMAS). Subsequently, the cultivation of Ling-zhi fruiting body was scaled up and the technique was published in *Compilation of Microbial Data*, and spread across the country (Anonymous 1971). Since then, the cultivation of Ling-zhi fruiting body has rapidly increased in China and other Asian countries such as Japan and Korea.

### 2.4. Processing

Based on the sufficient understanding of Ling-zhi species, resources and appreciable application potential, processing of Ling-zhi was then conducted with different commercial demands. In IMCAS, H.A. Wen developed different applications for Ling-zhi resources, including a method for breakage of Ling-zhi spore walls, extraction of Ling-zhi spore oil, and isolation of chitosan from the residues of Ling-zhi fruiting bodies after oil or polysaccharide extraction.

Generally, mechanical disruption of Ling-zhi spore walls results in high temperatures and oxidation, which significantly affects the spore powder quality. New techniques were developed to break spore walls with temperatures of −15 to 0°C, which ensured the effective protection of bioactive substances from oxidative damage and high temperatures. After optimizing the processing conditions, the breakage rate of spore walls could reach 95–99%. To extract Ling-zhi spore oil, CO_2_ supercritical fluid extraction was used. Commonly, Ling-zhi spore powder is granulated with an adhesion agent before extraction to avoid jamming of the device. Wen improved the extraction process by placing a piece of filter paper on the exit of the extraction device. This innovation ensured the protection of the machines from jamming and protected the oil from contamination by adhesion agents. The extraction rate could reach 29% with this patented technique (ZL200310121184.9), compared with the previous method, which was less than 20%. After extraction of water-soluble substances, fruiting body residues remain, which are rich in chitosan. Therefore, Wen designed an efficient and environment-friendly isolation process for isolating refined chitosan from the residue, yielding high purity white chitosan (deacetylation degree of 98%). This technique was also patented (ZL200910244636.X). These three processing techniques furthered the use of Ling-zhi resources and are used in some factories.

### 2.5. Chemical analysis and evaluation

Different bioactive components from *Ganoderma* spp. have been isolated and evaluated, such as polysaccharides (Pi et al. 2014; Zhang, Nie, et al. 2014; Zhu et al. 2016), triterpenoids (Zhang, Tao, et al. 2014; Peng et al. 2015; Smina et al. 2016), immune-modulating proteins (Guan et al. 2014; Lin et al. 2014), steroids (Ko et al. 2008; Seo et al. 2009), and alkaloids (Zhao et al. 2015; Huang et al. 2016). Scientists at IMCAS also conducted the chemical analysis and evaluation of components from interested *Ganoderma* species, so as to reveal their application potential. For example, H.W. Liu’s team isolated six novel triterpenes from *G. boninense* Pat., which showed antimalarial effects and agonistic activity to LXRβ after being evaluated. The result had provided evidence for the potential usage of *G. boninense* for the treatments of malarial diseases and metabolic symptoms (Ma et al. 2015). They also did the first chemical investigation on *G. leucocontextum* T.H. Li, W.Q. Deng, Dong M. Wang & H.P. Hu (白肉灵芝), which was recently described from Tibet Autonomous Region and Sichuan Province of China as a new species (Li et al. 2015). A variety of novel triterpenes have been isolated from *G. leucocontextum*, and most of them have potential anti-disease activities after being actively evaluated (Wang et al. 2015). This result has provided evidence for the usage of *G. leucocontexum* as a herbal medicine and a promising source of new bioactive agents.

## 3. Ling-zhi industry in China

With long history of myths worshipping, ‘Ling-zhi’ has been widely popularized and accepted by public. Artificial cultivation of ‘Ling-zhi’ fruiting body fascinated the scientific researches and industrially developed for utilization as medicine, tonic, and dietary supplements both in China and abroad. Over the past two decades, the industry has developed to offer over 780 products made from Ling-zhi that are commonly available in markets (http://app1.sfda.gov.cn/datasearch/face3/dir.html).

### 3.1. Ling-zhi cultivation

The large-scale cultivation of Ling-zhi fruiting bodies is conducted on logs or substrates in bags. Log cultivation uses the entire tree trunk, small logs, or tree branches as the main cultivation substrates. The advantages for producing fruiting bodies on logs include higher quality, greater active constituent contents, and larger spores. However, this method takes nearly six months to harvest the fruiting bodies, and the continuous consumption of wood is detrimental to the forestry. Bag cultivation uses agricultural waste, such as cottonseed hulls, corncobs, sawdust, and wheat bran as the main substrates. Ling-zhi hyphae grow faster in bag cultivation compared with log cultivation, and the fruiting bodies can be harvested quickly. However, bag substrate cultivation is generally considered to be lower in quality and risks contamination of heavy metals and pesticides from agricultural wastes.

The main species cultivated in China is called chi-zhi (赤芝) (known as ‘*G. lucidum*’ in most historical literatures). Other species, such as *G. sinense* J.D. Zhao, L.W. Hsu & X.Q. Zhang and *G. tsugae* Murrill are also cultivated, but their production is limited.

There are estimated to be 200,000 farmers working in the Ling-zhi cultivation in China, and there are about 200 Ling-zhi enterprises. Several Ling-zhi cultivation regions in China have been recognized, including: Northeast China (Jilin, Liaoning provinces), East China (Anhui, Jiangsu, and Zhejiang provinces), South China (Hainan, Fujian provinces), and Southwest China (Sichuang, Yunnan provinces). These regions are in accordance with the natural *Ganoderma* species distribution areas. Generally, Ling-zhi cultivation in Northwest China and Qinghai-Tibet Plateau is slower because of the special climatic conditions. Nevertheless, there have been some recent breakthroughs such as the successful cultivation of the novel species, *G. leucocontextum*, by the Academy of Agricultural Sciences, Tibet Autonomous Region (http://www.emushroom.net/news/201408/06/20258.html).

### 3.2. Ling-zhi mycelium fermentation

Cultivation of Ling-zhi fruiting bodies is time-consuming and product quality is not always consistent. For commercial benefit, a more economical, efficient, and controllable method is mass production of Ling-zhi mycelia using a fermentation tank in liquid media. Mycelia and different metabolites can be collected after only a few days and developed into different products. Fermentation of Ling-zhi mycelia is a promising alternative because it has a shorter production cycle, higher product yield, consistent quality, and a lower cost.

The two most valuable bioactive metabolites of Ling-zhi are polysaccharides and ganoderic acid (triterpenoids). During fermentation, the carbon source, nitrogen source, mineral salt, microelements, temperature, pH, time, and oxygen partial pressure are crucial factors for the yield of these substances. After optimization of the nutrients and fermentation conditions, biomass can be up to 16–23 g/L, intracellular polysaccharides to 2–4.7 g/L, and ganoderic acid to 496–798 mg/L (Tang & Zhong 2003; Chang et al. 2006; Xu et al. 2008; Tang et al. 2011).

### 3.3. Ling-zhi products

Ling-zhi products may be produced from Ling-zhi fruiting bodies, Ling-zhi spore powder, Ling-zhi fermentation mycelia, or fermentation broth. There are two types of Ling-zhi products: nutraceuticals and pharmaceuticals.

Various Ling-zhi nutraceuticals are available in the market, and 572 items can be found in the database of the China Food and Drug Administration (CFDA). As is officially confirmed by the CFDA, the healthcare functions of these products include tumour inhibition, immunoregulation, anti-fatigue, sleep promoting, anti-aging, blood sugar and blood fat regulation, and liver protection. The main forms of Ling-zhi nutraceuticals are intact Ling-zhi fruiting bodies, fruiting body slices, broken wall Ling-zhi spore powders, and Ling-zhi spore oil.

Intact Ling-zhi fruiting bodies are made from whole fresh fruiting bodies after being dried. These are used in crafts or ornaments because of their implication of happiness and good fortune. Fruiting body slices can be consumed in teas, or used in cooking to improve well-being and health in the daily diet. When the fruiting body matures, spores eject and drop on the pileus. Collected spores called spore powders are then broken to produce broken wall Ling-zhi spore powders. This product is regarded as the essence of Ling-zhi. Ling-zhi spore oil is extracted from the spores, but is expensive because of its low extraction rate of 15–20%.

Ling-zhi pharmaceutical products are mainly used as an adjuvant therapy for tumours or cancers. Two species, chi-zhi (赤芝) and zi-zhi (*G. sinense*, 紫芝), are recorded as legal medicinal fungi in ‘Pharmacopoeia of People’s Republic of China 2000 edition (Part one)’ (CPC 2000). The principle therapeutical effect of Ling-zhi is to enhance the phagocytosis or cell cytotoxicity of immune cells, such as macrophages, natural killer cells, T lymphocytes, and B lymphocytes (Lin 2005).

Other products include those derived from water extraction, ethanol extraction, or spore powder extraction; Ling-zhi make-up products; Ling-zhi wine; and mycelia capsule.

### 3.4. Ling-zhi market

A variety of Ling-zhi products have been commercialized and it is estimated that at least 100 brands are sold in the markets (Lai et al. 2004). Ling-zhi products, particularly in reference to nutraceuticals, are consumed as a dietary supplement, especially in Asian countries, and yearly sales of Ling-zhi products have reached US$2.5 billion (Chang 2004).

China is the biggest producer and exporter of Ling-zhi with a capacity over 110,000 ton/year. Large amounts of Ling-zhi products are exported every year, and are an important part of the foreign exchange earning potential of Chinese foods and medicinal fungi. In the domestic market, Ling-zhi fruiting bodies, Ling-zhi slices, and Ling-zhi spore powders are the three most popular products among consumers who are eager to strengthen their physical health or prevent and treat diseases.

## 4. Perspectives

Overall, Ling-zhi in China has gone from preliminary understanding of resources and culture, to realization of artificial cultivation, large-scale cultivation, isolation of bioactive components, exploitation of processed products, and creation of a Ling-zhi industry. The research results from IMCAS have provided essential support for the development of Ling-zhi and prompted the development of the Ling-zhi industry in China to some extent.

To reach the growing economic potential of the Ling-zhi industry, a number of challenges need to be met. First, taxonomic research into Ling-zhi species needs to be systematic. Incorrect scientific names of Ling-zhi species (other than chi-zhi) may still exist and need to be corrected. Second, standard Ling-zhi cultivation technologies are needed to meet market demand. Because of the development of Ling-zhi fruiting body cultivation, farmers can plant it after being trained. This has resulted in the majority of Chinese cultivators being individual workshops. However, suboptimal Ling-zhi products may be produced from these workshops, because of non-standard techniques, which could decrease the quality of Ling-zhi product in the market. Third, fermentation techniques still need to be optimized to increase compounds for different purposes.

Finally, despite the large market, there are still remaining problems in the Ling-zhi industry such as the homogeneity of the products, lack of high value-added products, low quality, and high prices. Herein, bioactive components need to be isolated and their potential function clarified, which could lead to diversified products. Meanwhile, an effective market order has yet to be built and improved.
